# VOSviewer-Based Bibliometric Network Analysis for Evaluating Research on Juvenile Primary Fibromyalgia Syndrome (JPFS)

**DOI:** 10.3390/children9050637

**Published:** 2022-04-28

**Authors:** Alessandro Vittori, Marco Cascella, Marianna Leonardi, Federica Monaco, Davide Nocerino, Arturo Cuomo, Alessandro Ottaiano, Francesco Perri, Ilaria Mascilini, Elisa Francia, Emiliano Petrucci, Franco Marinangeli, Sergio Giuseppe Picardo

**Affiliations:** 1 Department of Anesthesia and Critical Care, ARCO Roma, Ospedale Pediatrico Bambino Gesù, IRCCS, Piazza S. Onofrio 4, 00165 Rome, Italy; ilaria.mascilini@opbg.net (I.M.); elisa.francia@opbg.net (E.F.); sgiuseppe.picardo@opbg.net (S.G.P.); 2 Department of Anesthesia and Critical Care, Istituto Nazionale Tumori—IRCCS, Fondazione Pascale, Via Mariano Semmola, 53, 80131 Naples, Italy; m.cascella@istitutotumori.na.it (M.C.); monacofederica@gmail.com (F.M.); dr.nocerino@gmail.com (D.N.); a.cuomo@istitutotumori.na.it (A.C.); 3 Department of Anesthesia and Critical Care, University of Campania Luigi Vanvitelli, Viale Abramo Lincoln 5, 81100 Caserta, Italy; leonardi1994@gmail.com; 4 Division of Abdominal Oncology, Istituto Nazionale Tumori—IRCCS, Fondazione Pascale, Via Mariano Semmola, 53, 80131 Naples, Italy; a.ottaiano@istitutotumori.na.it; 5 Head and Neck Medical Oncology Unit, Istituto Nazionale dei Tumori—IRCCS, Fondazione Pascale, Via Mariano Semmola, 53, 80131 Naples, Italy; f.perri@istitutotumori.na.it; 6 Department of Anesthesia and Intensive Care Unit, San Salvatore Academic Hospital of L’Aquila, Via Vetoio, 48, 67100 L’Aquila, Italy; petrucciemiliano@gmail.com; 7 Department of Anesthesiology, Intensive Care and Pain Treatment, University of L’Aquila, Piazzale Salvatore Tommasi, 1, 67100 Coppito, Italy; francomarinangeli@gmail.com

**Keywords:** fibromyalgia, juvenile primary fibromyalgia syndrome, pain, chronic pain, bibliometric network analysis, children, pediatrics, musculoskeletal pain, childhood, adolescence

## Abstract

Background: Juvenile primary fibromyalgia syndrome (JPFS) is a chronic musculoskeletal pain syndrome that affects children and adolescents. Methods: A VOSviewer-based bibliometric network analysis was performed by scanning the global literature on JPFS in the Web of Science (WOS) online database. The search string applied to identify the closest matching articles was “juvenile primary fibromyalgia syndrome (all field)”. Results: A total of 67 articles on JPFS were published from 1985 to March 2022, in the WOS. Regarding article types, 39 were research manuscripts, 16 reviews, 8 meeting abstracts, 2 letters, 1 book chapter, 1 correction, and 1 proceeding paper. The Quartile analysis demonstrated that 44% of papers were published in Q1, 37% in Q2, 8% in Q3, and 11% in Q4. Conclusions: Our analysis highlights that more efforts are warranted to increase the production of quality papers and enhance the connections between the various research groups. JFPS represents a research field still to be explored and which deserves greater investments to obtain quality scientific evidence.

## 1. Introduction

Juvenile primary fibromyalgia syndrome (JPFS) is a chronic musculoskeletal pain syndrome that affects children and adolescents with an estimated prevalence that varies from 1.2 to 6.2% [1,2,3]. Clinical features are diffuse and persistent pain, sleep disturbance, fatigue, and multiple specific tender points on physical examination [4,5]. Despite a lot of research being conducted, the etiology and pathogenesis have not been entirely discovered [6,7]. Several contributing factors have been suggested; intrinsic factors include low pain thresholds, female gender, joint hypermobility, poor perceived control over pain, maladaptive pain-coping strategies, and difficult temperament; extrinsic factors encompass antecedent pain occurrences, physical or sexual abuse, sleep disturbance, and reduced fitness [8,9,10]. 

The are many aspects that make treating these children challenging. These issues mostly concern the inadequacy of validated diagnostic criteria and evidence-based consensus guidelines and a scarce understanding of the epidemiology and natural history of this clinical condition [11]. In addition to this, there are the developmental changes that occur during childhood and adolescence, as well as the cognitive and metacognitive competencies required for a child to give reliable self-reports of pain [12,13]. Finally, the way its chronic course can potentially affect the functional status and psychological development of these patients must be better investigated [14].

Usually, the clinical diagnosis is established only on the patient’s history and physical examination that demonstrate general tenderness (muscle, joints, and tendons) combined with the lack of other pathological findings able to justify pain and fatigue and normal laboratory tests [5]. These diagnostic limitations can lead to a sense of insecurity in healthcare professionals and induce unnecessary examinations and potentially dangerous drug prescriptions. Patients can undergo clinical evaluation from manifold pediatric specialists such as neurologists, rheumatologists, and other specialists before the correct diagnosis is reached [12,15]. 

The aim of this bibliometric analysis is to dissect the developed research on the subject, provide helpful findings, predict the direction of future studies, implement corrective measures, and improve research networks.

## 2. Materials and Methods

The global literature on JPFS was checked in the Web of Science (WOS) online database. The search string adopted to find the manuscripts with similarities for clustering was “juvenile primary fibromyalgia syndrome (all field)”. All data were acquired on 27 March 2022. Several features for the documents that satisfied the prerequisites were extracted. These features included authors (s), journal, year of publication, article type, topic, number of citations, and quartile. We identified 4 topics: diagnosis, pathogenesis, drug therapy, and non-drug therapy. An article could contain more than one topic.

The quartile analysis was used for evaluating the journal’s metrics. The journal’s quartile was calculated based on the journal’s position in its subject category (category ranking). It was configured as a position indicator: from the first (best) quartile or Q1 to the fourth (worst) quartile or Q4. The best Quartile was used if two or more categories were detected. The source was Journal Citation Reports™ 2020 (Clarivate Analytics). 

The literature analysis and knowledge visualization software tool VOSviewer (version 1.6.17) was used. This is a software tool used for the structuring and graphical visualization of bibliometric networks. Scientific interconnections can include journals, researchers (authors), or single publications and can be built based on citations, bibliographic couplings, co-citations, or co-authorship relationships. The tool also allows text extraction capabilities that can be used to build and visualize co-occurrence networks of important linguistic elements mined from a corpus of scientific literature. In this analysis, VOSviewer was used to analyze co-authorship for countries, co-occurrence of keywords (interconnection), co-citation (bibliographic coupling), and analysis of the most productive organizations and networks.

**Figure 1:**
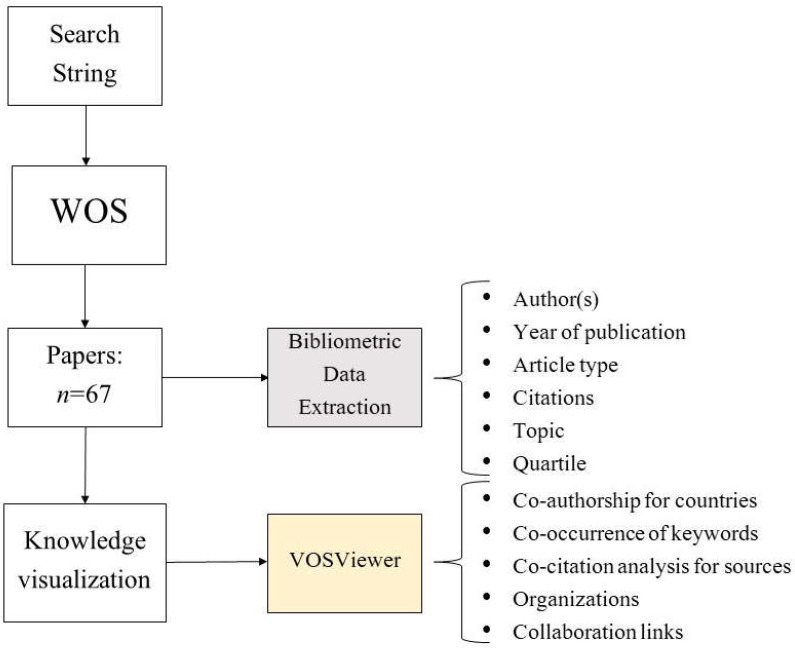
Flowchart of the study. Abbreviations: WOS, Web of Science.

The flowchart of the study is given in Figure 1.

## 3. Results

A total of 67 articles on JPFS were published from 1985 to March 2022, in the WOS. Regarding article types, 39 were research articles (35 observational studies, 2 randomized controlled trials, and 2 other kinds of studies), 16 were reviews (15 narrative review and 1 systematic review), 8 were meeting abstracts, 2 were letters, 1 was a book chapter, 1 was a correction, and 1 was a proceeding paper. The most relevant topics of the papers selected by our bibliometric analysis were “diagnosis” (*n* = 13) and “drug-therapy” (*n* = 9).

**Figure 2:**
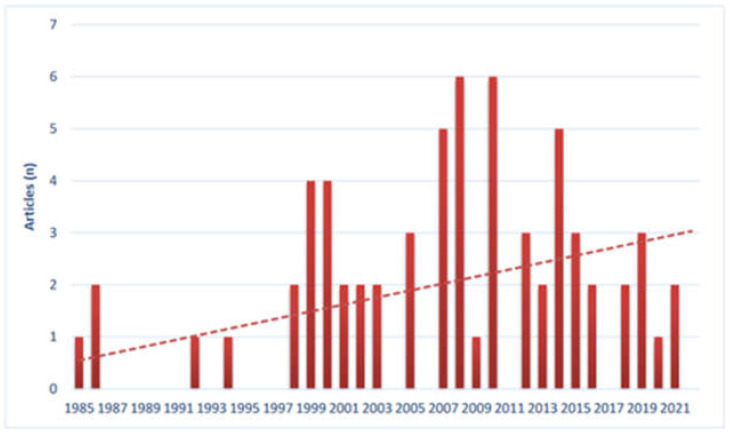
The annual number of documents and trend.

The annual number of documents and trend is shown in Figure 2. 

The most cited articles are reported in Table 1.

**Figure 3:**
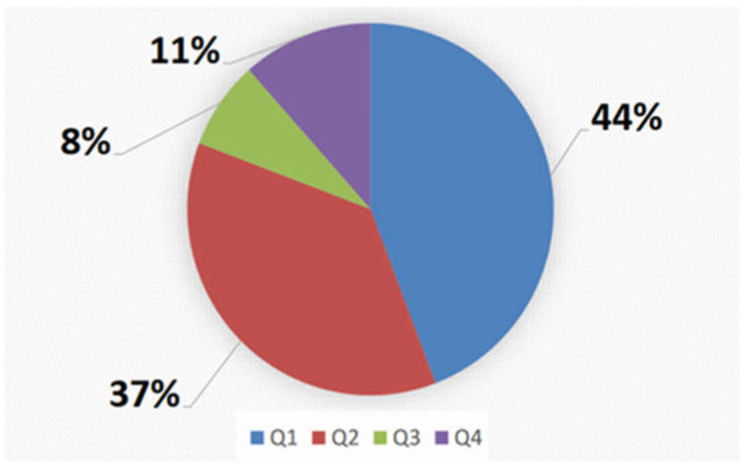
Quartile analysis.

The Quartile analysis demonstrated that 44% of papers were published in Q1, 37% in Q2, 8% in Q3, and 11% in Q4 (Figure 3).

### 3.1. Co-Authorship for Countries

By selecting a minimum number of documents of a country of 2, the software produced 6 countries, and 3 met the threshold. An amount of 40 documents and 1544 citations were obtained from the US (Total link strength: 2); 4 documents and 153 citations were obtained from Israel (Total link strength: 2). Finally, 2 documents and 132 citations (without links) were obtained from Germany. 

### 3.2. Co-Occurrence of Keywords

**Figure 4:**
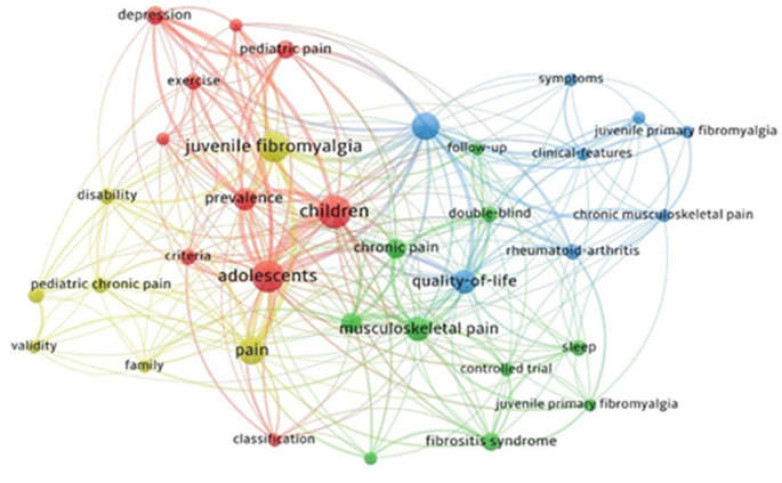
Co-occurrence of keywords. The size of nodes illustrates the frequency of occurrence. The curves between the nodes indicate their co-occurrence in the same publication. The shorter the distance between two nodes, the larger the number of co-occurrences of the two keywords. The analysis yielded 240 keywords; of those, 35 satisfied the threshold (3 times), and finally, 4 clusters were obtained (different colors).

This investigation focused on the keywords of the articles. We established use the keywords detected more than 3 times in the WOS core dataset. Of the 240 keywords screened, 35 met the threshold. The keywords that appeared most were ADOLESCENTS (occurrence: 13; total link strength: 71); CHILDREN (14/68), and JUVENILE FIBROMYALGIA (12/56) (Figure 4).

### 3.3. Co-Citation Analysis for Sources

**Figure 5:**
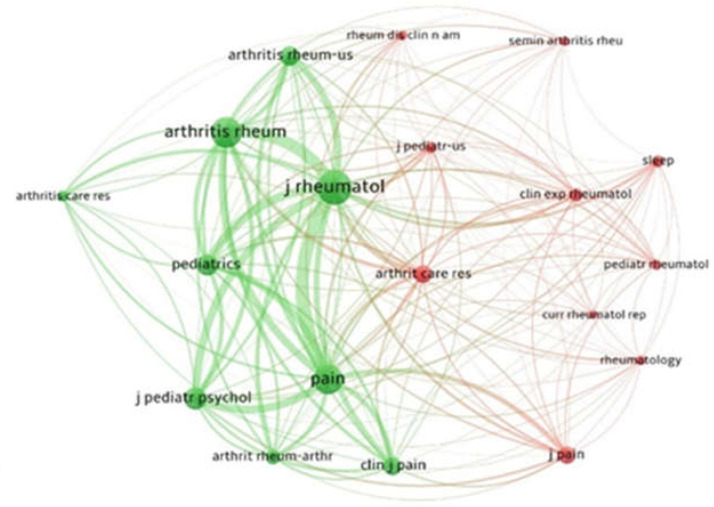
Co-citation analysis for sources. The lowest number of citations for a source was *n* = 17. Of the 597 sources, 19 satisfied the threshold.

For the co-citation analysis, we adopted the cited sources (i.e., journals) as a unit of analysis. The lowest number of citations of a source was 17. Thus, of 597 sources, 19 satisfied the threshold. For each source, we measured the aggregate strength of the co-citation links with other sources. The Journal Rheumatology collected 165 citations and 5905 links; PAIN collected 124 citations and 4777 links; Pediatrics collected 72 citations and 3193 links (Figure 5).

### 3.4. The Most Productive Organizations and Their Collaborations 

**Figure 6:**
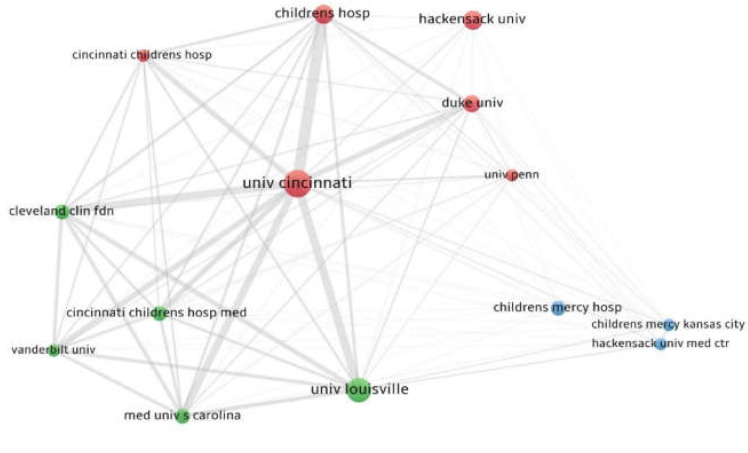
The most productive organizations and collaboration links.

The study of affiliations (organizations) was carried out by accounting for the cut-off of 3 as the lowest number of papers. Out of 60 organizations, 14 satisfied the threshold. For each of these 14 organizations, the total strength of the citation links with other organizations was calculated. The organizations with the greatest total link strength were selected. The University Of Cincinnati, Cincinnati, OH (USA) published 11 documents and a total link strength of 6919; The University Of Louisville, Louisville, KY, USA published 9 documents and 1548 collaboration links; The Children Hospital published 6 documents and 1489 links (Figure 6).

## 4. Discussions

A bibliometric analysis is a useful tool for clarifying research in fields where there are scientific gaps and a consensus about diagnosis and/or treatment [16,17,18]. The software tool VOSviewer was used for a bibliometric analysis on several arguments, such as COVID-19 [19], oncology [20], pain medicine [16], and others. On these premises, our intent was to highlight the gaps in fibromyalgia research, especially in its form that affects children and adolescents.

Fibromyalgia is a multifactorial pathology, whose diagnostic–therapeutic step is difficult: diagnosis, differential diagnosis, therapy, and follow-up [21]. Similarly to what is observed in other types of chronic pain, these difficulties are magnified when dealing with pediatric patients [22]. In fact, in this setting, clinical management and, above all, research purposes are difficult to perform. In the pediatric field, difficulties in patients’ recruitment and the limited number of pediatric hubs for pain therapy are serious obstacles in chronic pain investigations.

Our analysis underlines that JPFS is a topic of great interest, with an increase in related publications over time. However, the scientific corpora of publications on the topic has several limitations. The first and obvious is that more articles come from a limited number of countries. It is a clear sign that research in this field requires an important effort. Furthermore, collaborations are scarce, making the results limited to a few centers. Nevertheless, the selected articles were published mostly in Q1 (44%) and Q2 (37%) journals. Probably, the possibility of publication in prestigious journals testifies that JPFS is of great interest and topicality. On the other hand, musculoskeletal pain is extremely represented in chronic pain [23]. 

## 5. Limitations

The string we used for bibliometric analysis [“juvenile primary fibromyalgia syndrome (all field)”] produced a limited number of articles, compared to the number of articles potentially obtainable with less restrictive search strings. Although this choice excluded a considerable part of literature, on the one hand, it had the advantage of highlighting that the syndrome is poorly characterized. In fact, clinicians and researchers often refer to the pediatric population through the clinical and diagnostic aspects of adult fibromyalgia. However, the literature underlines that a well-defined syndrome exists and deserves the right attention from the scientific world. Our analysis demonstrates that there are obvious gaps in this research area.

## 6. Conclusions

JPFS is chronic musculoskeletal pain disease affecting children and adolescents. It represents a major social problem and a challenge for researchers. Our analysis highlights that more efforts are needed to increase the production of high-quality papers and increase the connections between the various research groups.

## Figures and Tables

**Table 1:** Summary of the most cited articles. Abbreviations: Q, Quartile.

Autor(s)	Article Title	Journal	Quartile	Citations (*n*)	Year
Yunus, MB; et al.	Juvenile primary fibromyalgia syndrome—a clinical-study of 33 patients and matched normal controls	Arthritis and rheumatism	Q1	211	1985
Abad, VC; et al.	Sleep and rheumatologic disorders	Sleep medicine reviews	Q1	196	2008
Schanberg, LE; et al.	Daily pain and symptoms in children with polyarticular arthritis	Arthritis and rheumatism	Q1	137	2003
Herrmann, M	Stress and rheumatic diseases	Rheumatic disease clinics of North America	Q4	126	2000
Kashikar-Zuck, S; et al.	Depression, coping, and functional disability in juvenile primary fibromyalgia syndrome	Journal of pain	Q1	121	2021
Campo, JV	Annual Research Review: Functional somatic symptoms and associated anxiety and depression—developmental psychopathology in pediatric practice	Journal of child psychology and psychiatry	Q1	119	2012
Kashikar-Zuck, S; et al.	Cognitive behavioral therapy for the treatment of juvenile fibromyalgia: A multisite, single-blind, randomized, controlled clinical trial	Arthritis and rheumatism	Q1	107	2012
Coles, ML; et al.	Juvenile primary fibromyalgia syndrome: A Review- Treatment and Prognosis.	Pediatric rheumatology	Q2	111	2021
Ghaly, MS; et al.	Social functioning and peer relationships of adolescents with juvenile fibromyalgia syndrome	Arthritis and rheumatism	Q1	90	2007
Kashikar-Zuck, S; et al.	Family Factors, Emotional Functioning, and Functional Impairment in Juvenile Fibromyalgia Syndrome	Arthritis & rheumatism-arthritis care & research	Q1	86	2008

## Data Availability

The data can be consulted from the corresponding author for a reasonable reason.
